# An Overview of the Development of New Vaccines for Tuberculosis

**DOI:** 10.3390/vaccines8040586

**Published:** 2020-10-05

**Authors:** E. Whitlow, A. S. Mustafa, S. N. M. Hanif

**Affiliations:** 1 Department of Basic Sciences, Kentucky College of Osteopathic Medicine, University of Pikeville, Pikeville, KY 41501, USA; emmawhitlow@upike.edu; 2 Department of Microbiology, Faculty of Medicine, Kuwait University, Safat 13110, Kuwait; abusalim@hsc.edu.kw

**Keywords:** vaccines, *Mycobacterium tuberculosis*, tuberculosis, BCG, new vaccines

## Abstract

Currently, there is only one licensed vaccine against tuberculosis (TB), the Bacillus Calmette–Guérin (BCG). Despite its protective efficacy against TB in children, BCG has failed to protect adults against pulmonary TB, lacks therapeutic value, and causes complications in immunocompromised individuals. Furthermore, it compromises the use of antigens present in the purified protein derivate of *Mycobacterium tuberculosis* in the diagnosis of TB. Many approaches, e.g., whole-cell organisms, subunit, and recombinant vaccines are currently being explored for safer and more efficacious TB vaccines than BCG. These approaches have been successful in developing a large number of vaccine candidates included in the TB vaccine pipeline and are at different stages of clinical trials in humans. This paper discusses current vaccination strategies, provides directions for the possible routes towards the development of new TB vaccines and highlights recent findings. The efforts for improved TB vaccines may lead to new licensed vaccines capable of replacing/supplementing BCG and conferring therapeutic value in patients with active/latent TB.

## 1. Introduction

Tuberculosis (TB), a disease caused by *Mycobacterium tuberculosis* (Mtb), is the leading cause of death due to an infectious agent in the world [1]. Mtb infection causes active TB disease in 10 million people and leads to 1.5 million deaths annually [1]. Furthermore, about a quarter of the world’s population (about 2 billion people) are latently infected with TB, which becomes a pool for active TB cases later in life [2]. Currently, there is only one licensed vaccine against TB, i.e., *Mycobacterium bovis* Bacillus Calmette–Guérin (BCG) [3], and it is one of the most widely used vaccines globally [4]. Each year, BCG is given to about 100 million children worldwide [5]. Vaccination with BCG prevents the most severe forms of childhood TB by reducing the risk of meningeal and miliary TB by 85% and TB-associated deaths by 66% [6]. The BCG vaccine protects children against TB for up to 10 years, but its efficacy declines with each subsequent year [7]. Additionally, it does not provide protection against adult pulmonary TB [8] and lacks any additional benefit upon revaccination of healthy and active TB cases [8]. Furthermore, there are safety issues related to the BCG vaccine, as it is a live attenuated vaccine with a risk of becoming virulent [8]. The BCG vaccine was first produced in 1921, and because of its shortcomings, a more effective and safer vaccine is needed to replace or supplement it [9].

Furthermore, another worldwide epidemic, type 2 diabetes, is associated with a 2–4 times increased risk of developing active TB [10], and a known glucose control medication lowers the immune response to exacerbate the risk further [11]. This, along with the emergence of drug-resistant TB, has increased the necessity for developing a better vaccine for TB [9]. In addition, an effective vaccine against TB may also protect against asthma, another non-infectious disease of high global prevalence [12].

Many vaccines are in the pipeline (Tables 1–3) with an aim to produce a new vaccine for TB, and different strategies include replacing BCG, boosting BCG, and therapeutic use [13]. Different approaches are explored as pre- and post-exposure vaccines for TB. This paper aims to give an overview of the TB vaccine candidates in the pipeline, discusses current vaccination strategies, and highlights recent findings.

## 2. Status of Current TB Vaccine: BCG

The only currently licensed vaccine against pulmonary and extrapulmonary TB is the live attenuated BCG vaccine. It was originally derived from the pathogenic *Mycobacterium bovis* that causes TB primarily in cows [14]. To attenuate its pathogenicity, two French scientists, Calmette and Guerin, cultured *M. bovis* on an artificial medium in vitro for 13 years (1908–1921) and a total of 231 passages. The resulting organism, BCG, did not cause TB lesions in various animal models but was immunogenic and protected the immunized animals against challenges with *M. bovis* [3]. Since 1921, the BCG vaccine has been used in infants and children and is effective in providing protection against childhood TB [15], avoiding 120,000 childhood deaths per year [16]. However, it fails to protect against adult pulmonary TB, the major manifestation of the disease [8]. For example, although high-incidence nation China has an overwhelming BCG vaccine coverage, three-quarters of TB is estimated to occur in the 45-year-old or older population [16]. BCG’s decline in efficacy after 10 years is an additional problem for countries with a high incidence of TB, as BCG vaccination occurs at birth, leaving people at risk later in life when they are most vulnerable [17]. In fact, the elderly have been shown to have a higher prevalence of TB across all age groups [9].

The BCG vaccine also poses a risk to immunocompromised individuals [18,19]. Those with primary immunodeficiencies are prone to develop severe vaccine-derived complications [18]. Additionally, BCG vaccination of HIV-exposed but uninfected infants resulted in reduced BCG-specific T-cell proliferation and production of lower concentrations of protective cytokines IFN-*γ* and TNF-*α*, compared to healthy infants [19]. The results further showed a delay in immune system maturation of HIV-exposed uninfected infants and hence resulted in a higher risk for developing TB [19]. Furthermore, BCG vaccination of HIV-infected infants and children has the risk of causing a TB-like disseminated disease known as BCG-osis [3]. In addition, BCG is a preventative pre-exposure or prophylaxis vaccine and is not effective as a therapeutic vaccine against post-exposure active and latent TB [14].

BCG also poses a complication for the diagnosis of active as well as latent TB by using the antigenic preparation of Mtb known as purified protein derivative (PPD) in Mantoux or tuberculin skin test (TST). In this test, PPD is injected intradermally to evaluate cell-mediated immunity in response to Mtb antigens. The intradermal injection of PPD produces a type IV hypersensitivity reaction, and is used for diagnosis of both active and latent TB [20]. Although the World Health Organization has recommended the use of this test for diagnosing tuberculosis in low-resource settings [21], BCG vaccination compromises PPD’s diagnostic value due to low specificity. This is because PPD is a crude mixture of several hundred proteins of *M. tuberculosis* and many of them are present in BCG as well, leading to antigenic cross-reactivity and causing a high rate of false positivity [22]. A reduction in the antigen cross-reactivity is required for test accuracy; however, limited research and discussions have addressed this problem.

## 3. Immune Characteristics and Markers for Mtb

The complexities of Mtb infection in humans create challenges in finding targets of the immune reaction to the pathogen. Mtb bacilli first enter the lungs in aerosol droplets. Due to the innate immune response, macrophages ingest the bacteria and recruit other immune cells [8]. Neutrophils, Delta T cells, NK cells, and CD4^+^ T cells are activated to control the infection, leading to the formation of a granuloma [9]. The granuloma contains a small number of bacilli that may contribute to the reactivation of disease later in life [23]. In the majority of cases, CD4^+^ T cells will control the bacteria from further infection; however, in some cases, the infection can reactivate and develop to an acute, chronic, or extrapulmonary infection [9]. The disease is efficient in evading the immune response and can be in the latent stage for years before symptoms are expressed [9]. Reasons why most people exposed to Mtb do not develop TB is not well understood [13].

The cytokines IL-12, IL-8, IFN-*γ*, TNF-*α*, TGF-β, IL-17, and memory T cells have been shown to be active in the immune response against TB [24]. While these markers are known to be involved in the immune response, Mtb does not have a known validated immune correlate of protection (COP). Without this, it requires years to collect data for study endpoints [13]. Satti and McShane [25] stated that COP would allow vaccines to be optimized and explored based on their match with the proven immune response, leading to licensure of vaccines. The COP could help explain the vaccine MVA85A’s dismal response in phase 2. In previous clinical trials, the vaccine produced high levels of CD4^+^ T cell response but did not produce a robust response in the phase 2 trial [26]. Although CD4^+^ T cells play a predominant role in protective TB immunity, strategies that boost CD8^+^ function also enhance vaccine efficacy [26]. In a nonhuman primate model of tuberculosis disease, depletion of CD8^+^ T cells in immunized monkeys led to reduced protection. Similarly, CD8^+^ T cell depletion in *M. tuberculosis*-infected and then antibiotic-treated monkeys led to increased susceptibility to reinfection, indicating their importance in conferring immunity in a vaccination or natural infection setting. Human CD8^+^ CCR7^–^ CD45RA^+^ effector memory T cells exhibit significant anti-mycobacterial activity [26].

## 4. Vaccine Approaches

Based on the current understanding of Mtb, there are a number of strategies for the application of new vaccines, i.e., preventive pre-exposure (for uninfected individuals/infants), preventive post-exposure (for latent TB/adolescents and adults), and therapeutic (for active TB) [27]. Pre-exposure vaccines either aim to induce a more robust protective immune response than BCG in order to prevent disease or produce a faster response, so the infection is not established [8]. The post-exposure vaccine strategy aims to either induce a robust and long-lasting response to prevent disease reactivation or eliminate latent TB by inducing sterilizing immunity so that bacteria are not able to be reactivated later and lead to active TB [8]. The only currently licensed vaccine, BCG, follows the preventative pre-exposure approach. An additional approach for categorizing vaccines in preclinical and clinical trials places vaccines in their biochemical forms: live attenuated, inactivated, adjuvanted protein subunit, and recombinant. Some vaccines have also been created as a boost to BCG [28,29], and others have used unique modes of administration in an attempt to boost immune response [30].

There are currently at least 23 vaccine candidates in clinical trials in humans, as shown in Table 1 and Table 2. The recent developments focus on live attenuated, inactivated, subunit and recombinant vaccine types; and various antigens and adjuvant combinations are being tested in the case of subunit vaccines.

## 5. Live Attenuated Vaccines

The vaccines using the live attenuated approach contain whole Mtb in its weakened or altered form [33]. MTBVAC is the only live attenuated vaccine undergoing clinical trials in humans, and currently, two trials for the vaccine are recruiting for phase 3 (NCT03767946; NCT03152903) (Table 1). This vaccine is designed as a replacement for BCG and as an immunotherapeutic agent [13]. This vaccine is based on one of the seven phylogenetic branches (L1–L7) of the *M. tuberculosis* complex (MTBC) known as lineage 4 (L4). MTBVAC is derived from a clinical *M. tuberculosis* isolate Mt103 after deletion of two virulence-related genes *phoP* and *fadD26*. Safety studies in immunocompromised mice with severe combined immunodeficiency (SCID) have shown that MTBVAC is more attenuated than BCG [34]. Preclinical studies have also been completed with attenuated *M. tuberculosis* belonging to two other strains of MTBC i.e., L2 (MTBVAC-L2) and L3 (MTBVAC-L3), two of the three modern lineages of *M. tuberculosis*. The study demonstrated that vaccination with MTBVAC, MTBVAC-L2, and MTBVAC-L3 had similar or superior protection compared to BCG in immunocompetent mice, when the immunized mice were challenged with the three representative strains. This study shows the potential of MTBVAC to protect against the globally diverse strains of MTBC [34]. MTBVAC has also been used as a vector to make a dual TB-HIV vaccine known as MTBVAC.HIVA^2auxo^ [35]. In this study, a recombinant strain was constructed by inserting HIV clade-1 A immunogen HIVA into the parental strain MTBVAC. This TB-HIV vaccine provided similar protective efficacy to the parental MTBVAC strain against Mtb challenge in mice. Additionally, the study showed an increased safety profile of MTBVAC.HIVA^2auxo^ in comparison with BCG and MTBVAC [35], showing promise for immunosuppressed individuals who are at risk of severe infection or death due to unregulated pathogen replication [32].

## 6. Inactivated Vaccines

Inactivated vaccines are often considered safer than live vaccines since they do not contain any infectious particles. However, immunogenicity is much weaker than live vaccines and may require multiple doses [32]. These vaccines have been used for both pre- and post-exposure strategies [27]. Among the inactivated vaccines is RUTI, which contains detoxified and fragmented *M. tuberculosis* cells in liposomes (Table 1). The strategy of inducing an immune response and protection by inactivated organisms belonging to non-pathogenic and environmental mycobacterial species is also in clinical trials, including *M. vaccae*, *M. Indicus pranii* (MIP), and *M. obunese* (DAR-901) (Table 1).

A phase 2b vaccine, RUTI, has exhibited significant humoral and cellular immune responses against antigens expressed in actively growing and latent bacilli [36]. It has shown efficacy in controlling latent TB in experimental animals, i.e., mice, goats, guinea pigs and mini pigs [37], and the RUTI and *M. vaccae* are being used as therapeutic vaccines to reduce drug treatment duration for patients with active TB [36,38,39].

A recent study, using an ex vivo mycobacterial growth inhibition assay (MGIA), has shown RUTI vaccine-induced inhibition in mycobacterial counts and a significant shift towards Ly6C^-^ monocyte phenotype in the spleens of immunized mice [40]. Additionally, the vaccination of mice with RUTI upregulated the expressions of Ly6C^-^ related mRNA transcripts in splenocytes, producing a monocyte phenotype shift from LyC6^+^ [40]. Ly6C^-^ monocytes have been shown to have an anti-inflammatory role [41], whereas Ly6C^+^ monocytes are proinflammatory [41]. Therefore, the RUTI vaccine may show a more balanced immune response.

## 7. Subunit/Adjuvanted Vaccines

In comparison to whole pathogen vaccines, subunit vaccines contain selected parts of the pathogen. While this is generally a safer approach, it can produce a less robust immune response [32]. Therefore, to increase efficacy, subunit vaccines are often administered with an adjuvant [8]. Subunit/adjuvanted vaccines are an active area of research (Tables 2 and 3) and combinations are often noted as antigen:adjuvant. Since Mtb expresses around 4000 proteins [24], there are many possibilities for immune targets. Common secreted proteins used in subunit vaccines include ESAT6, MPT64, Ag85B, and Ag85A because of their different expression profiles with those either actively infected with TB or with latent TB [24]. Other proteins identified with high immunogenicity include Rv1031, Rv1198, Rv2016, Rv3874, Rv3875, Rv2031c [42,43], Rv3620 [44], PPE39 [45], and A60 complex [46]. Mtb antigens are expressed at different stages of infection; for example, ESAT-6 is always expressed, but Ag85B is mainly expressed early in infection [47]. This poses a challenge for creating a vaccine that produces enough cell stimulation. H56 is an example of a protein fusion that stimulates immune responses to antigens expressed at different stages of Mtb infection. It is a combination of Ag85B, ESAT-6, and Rv660c. In a recent preclinical trial, the vaccine H56: CAF01 has shown activation of both innate and adaptive immunity in mice [48]. This study and others showed hope for new subunit delivery strategies [49].

Aluminum-based adjuvants are primary adjuvants used in vaccines to drive a humoral response. Humoral immunity is not considered to have a major role in protection against Mtb, as it is an intracellular pathogen [9], therefore novel adjuvants may be useful to induce protective Th1 responses [50]. Current adjuvants used in Mtb vaccines include IC31, GLA-SE, AS01E, QS21, CFA01, and DPC [24].

An exciting study with subunit vaccine H4: IC31 was performed in adolescents in a high-risk setting for TB to assess its efficacy in reducing *M. tuberculosis*-specific immune response (an indicator of active TB) in individuals immunized with BCG in neonatal age [51]. The results were compared with BCG revaccination. All the participants had negative results for *M. tuberculosis*-specific immune response as determined by quantifying the concentration of IFN-*γ* secreted by using the QuantiFERON-TB Gold In-tube assay (QFT). QFT conversion, shown by the authors as having a higher risk of progression to TB, was determined in both H4: IC31 vaccinated group and BCG revaccinated group. BCG revaccination reduced the rate of QFT conversion (with an efficacy of 45% and 95% CI 6-68) compared to the H4: IC31 (efficacy of 31%, 95% CI 16–58) [51]. This study renewed interest in the revaccination with BCG, which had previously been accepted as having no effect [7,14].

In another study, a phase 2b clinical trial, the subunit vaccine M72/AS01E prevented pulmonary TB in adults already infected with Mtb with an efficacy of 54% [52]. In human studies with six candidate TB vaccines, i.e., MVA85A, AERAS-402, H1: IC31, M72/AS01E, ID93 + GLA-SE, and BCG, M72/AS01E was found to induce higher memory Th1-cytokine expressing CD4^+^ T-cell memory responses [53]. The ability of M72/AS01E to induce the highest memory CD4^+^ T cell response demonstrates the best vaccine take because the induction of these cells correlates with protective immunity in TB [53].

## 8. Recombinant Vaccines

Recombinant vaccines are produced using the techniques of genetic engineering. In brief, a piece of DNA encoding an antigen of Mtb is inserted into an appropriate vector to transform either a bacterial, mammalian, or yeast cell. The recombinant vector produces the antigen in large quantities in those cells [54]. Because the recombinant antigen can also be purified, the purified antigen can be used as a subunit vaccine to stimulate the immune response and avoid some of the potential concerns of other types of vaccines, such as whole-cell vaccines [55].

Recombinant vaccines (Table 2 and Table 3) can be placed into groups based on the type of organism used to express Mtb antigens: live mycobacterial, live bacterial, and live viral vaccines. In live mycobacterial vaccines, bacteria such as *M. bovis* BCG, *M. vaccae*, and *M. smegmatis* have been used as vectors. *M. bovis* BCG is commonly used as an expression vector due to its stability, cost-effectiveness, and non-specific immune stimulation [36,56,57]. Another recombinant bacterial vaccine is Pnz8149-ag85a/NZ3900 which uses *L. lactis* as a vector. This recombinant vaccine is at a preclinical stage, and it was able to induce both cellular and antibody responses after mucosal immunization in mice [58]. Many other recombinant bacterial vaccines are in preclinical stages (Table 3).

Several live viral-vector based vaccines have also entered into clinical trials (Table 2). The MVA85A vaccine, also known as AERAS-485, is currently being evaluated in two phase 2a trials (Table 2). One other vaccine is in phase 2a, and five vaccines are currently being evaluated in phase 1 studies (Table 2). In general, live viral-vector based vaccines have advantages for safety and ease of production but have the disadvantage of gene expression instability [32].

## 9. Recombinant Vaccine Candidates Using *M. tuberculosis*-Specific Antigens

The above stated recombinant DNA vaccines undergoing clinical trials are based on cross-reactive antigens of *M. tuberculosis*. These antigens may have the same problem of false positivity as BCG, with respect to the diagnosis of TB using PPD. Furthermore, because of the cross-reactivity with environmental mycobacteria, these candidate vaccines may face the problem of masking or blocking effects and may not be useful as booster vaccines in BCG pre-vaccinated individuals [67]. According to the masking hypothesis, early sensitization with environmental mycobacteria confers some level of protection against TB that masks the effect of a vaccine given later in life due to the presence of cross-reactive antigens. The blocking hypothesis postulates that previous immune responses to cross-reactive antigens, because of environmental mycobacteria sensitization, prevents the taking of a new vaccine by efficient clearance of the new vaccine antigens [67]. The use of *M. tuberculosis*-specific antigens as new TB vaccines may overcome these problems of blocking or masking. Therefore, to have TB vaccines better than BCG, development of new subunit and/or recombinant vaccines based on *M. tuberculosis*-specific antigens is required and is currently in progress [68]. Although three *M. tuberculosis*-specific regions known as regions of differences (RD) were identified using classical molecular and biochemical techniques, the identification of all *M. tuberculosis*-specific regions was possible with advances in whole genome sequencing and the comparative genome analysis of *M. tuberculosis* with other mycobacterial genomes [69]. These RDs are deleted/absent in all BCG sub-strains currently under use in different parts of the world [69]. The computer-based analysis for open reading frames has suggested that these RDs can potentially encode a large number of antigenic proteins [70]. To identify the candidate proteins suitable for vaccine development, these proteins were first tested for their ability to induce cellular immune responses in vitro with cells from naturally infected animals and humans. Such experiments with peripheral blood cells from humans and cattle identified six antigens inducing strong cellular immune responses i.e., PE35, PPE68, ESAT-6, CFP10, Rv3619c, and Rv2346c [71,72]. Testing of these antigens in animal models of TB have shown that all of these antigens are capable of inducing Th1 responses when used in combination with appropriate adjuvants and delivery systems such as DNA vaccine vectors and non-pathogenic mycobacteria, including BCG [73,74,75,76,77]. In mice and guinea pigs, immunizations with ESAT-6, CFP-10, and Rv3619c resulted into protection against challenges with *Mtb* [78,79,80].

In humans, a recombinant subunit TB vaccine candidate, GamTBvac, which contains a dextran-binding domain modified Ag85a and *M. tuberculosis*-specific antigens ESAT6-CFP10 with an adjuvant has been tested in a phase I clinical trial [81]. Safety and immunogenicity of GamTBvac were determined in healthy volunteers previously vaccinated with BCG. The GamTBvac reached an acceptable safety profile and was tolerated well. Furthermore, the immunization protocols led to a significant increase in the concentration of the protective Th1-cytokine IFN-*γ* and IgG antibodies. The immune responses were induced to all three antigens included in GamTBvac [81]. This vaccine has moved to a phase 2a clinical trial in humans (Table 1). Another recombinant bacterial vaccine in the preclinical stage and based on *M. tuberculosis*-specific ESAT-6 antigen is *L. lactis* FnBPA+ (pValac:ESAT-6). When delivered by mucosal route, this vaccine produced a systemic Th1 cell response and significant increase in specific secretory immunoglobulin A production in colon tissue and fecal extracts [82]. In a booster model of mice preimmunized with BCG, this vaccine induced a significant increase in proinflammatory cytokines IL-17, IFN-*γ*, IL-6, and TNF-*α* from spleens of immunized mice [65]. These findings are novel because they represent the first successful step towards the development of an oral booster to the BCG vaccine by using a *M. tuberculosis*-specific antigen with a bacteria *L. lactis* as a DNA delivery system.

## 10. Routes of Vaccine Administration

BCG is administered intradermally, where it induces a strong systemic response but weak mucosal immune response [83]. This presents a challenge since Mtb is transmitted via the respiratory route. Although the effects of different routes of administration are not readily discussed in the literature, some studies have shown that an alternative route may be a better possibility for the administration of Mtb vaccines. The oral vs intradermal administration of BCG was evaluated in a small trial, and the results showed that oral BCG produced a stronger mucosal response in bronchoalveolar lavage cells in nasal washes and tears, although intradermal BCG produced a stronger response in the blood [84]. In another study, intravenous administration of BCG vaccines in rhesus macaques produced an increased T cell response and induced a large population of T cells in the lungs, showing much protective promise [85]. Additional routes used in subunit vaccine administration include subcutaneous and intranasal [83], edible, mucosal, and nanoparticle-based vaccines [9]. Edible-based strategies employ the use of antigens expressed in plants such as carrot, potato, tobacco, and *Lemna minor* to activate the immune response [9], and nanoparticle strategies use nanoparticles conjugated with antigens such as Ag85B to increase Th1 responses in lymphoid organs against Mtb [9]. However, an optimal route for Mtb vaccine administration is yet to be identified. Hence, additional work is required to identify a route of vaccine administration that provides maximum protection against infection with Mtb.

## 11. Summary

TB is an ancient disease with worldwide prevalence, but only one licensed vaccine, BCG, is available for use with many concerns. Hence, sincere efforts are being made globally for the development of improved vaccines against TB. Among the strategies to develop new TB vaccines, whole-cell mycobacteria, subunit, and recombinant vaccine strategies have been employed. These efforts have led to the identification of about two dozen vaccine candidates currently in the pipeline at different stages of clinical trials in humans. Furthermore, additional TB vaccine candidates are at the preclinical stage and are being tested in animal models of TB. The progress made for the development of improved vaccines against TB is highly encouraging and it is hoped that new licensed TB vaccines for preventive and therapeutic applications in humans may become a reality in the near future.

## Figures and Tables

**Table 1:** Live attenuated, inactivated, and subunit/adjuvant vaccines in clinical trials.

Vaccine	Vaccine Type	Phase	NCT Number or Author
MTBVAC	Live attenuated	Two trials recruiting for phase 3	NCT03767946; NCT03152903
RUTI	Inactivated	Phase 2a not yet recruiting	NCT02711735
*M. Vaccae* based	Inactivated	Two phase 3 trials	NCT01977768; Bourinbaiar, A.S., et al. [31]
*M. indicus pranii* (MIP)	Inactivated	Two phase 3 trials	NCT00265226; NCT00341328
*M. smegmatis*	Inactivated	Phase 1	Gong, W., et al. [32]
DAR-901	Inactivated	Phase 2a	NCT02712424
M72/AS01E	Subunit/adjuvanted	Phase 2b completed	NCT01755598
H4:IC31 (AERAS 404)	Subunit/adjuvanted	Phase 2b	NCT02075203
H56:IC31	Subunit/adjuvanted	Phase 2a ongoing; phase 2b recruiting.	NCT03512249
ID93 + GLA-SE	Subunit/adjuvanted	Phase 2a	NCT02465216
*GamTBvac*	Subunit/adjuvanted	Phase 2a	NCT03878004
AEC/BCO2	Subunit/adjuvanted	Phase 1 recruitment completed; Phase 2b	NCT03026972; NCT04239313
Mtb72F/AS02	Subunit/adjuvanted	Phase 2b	NCT00397943

**Table 2:** Recombinant vaccines in clinical trials.

Vaccine	Vector Type	Phase	NCT Number or Author
VPM1002	Recombinant mycobacterial	Phase 2 completed, recruiting for phase 3; phase 2/3	NCT04351685; NCT03152903
Aeras-402	Recombinant mycobacterial	Two phase 2b trials	NCT02414828; NCT01198366
rBCG30	Recombinant mycobacterial	Phase 1; preclinical in mice	Yadav, J., et al. [9] Kaushik, A., et al. [59]
MVA85A/AERAS-485	Recombinant live viral vectored	Two phase 2a trials completed	NCT00953927; NCT01151189
Ad35/AERAS-402	Recombinant live viral vectored	Phase 1 recruiting	NCT01683773
Ad5Ag85A	Recombinant live viral vectored	Phase 1 recruiting	NCT02337270
TB/FLU-04L	Recombinant live viral vectored	Phase 2a	NCT02501421
ChAdOx1.85A	Recombinant live viral vectored	Phase 1	NCT04121494
ChAdOx1.85A + MVA85A	Recombinant live viral vectored	Phase 1	NCT03681860
ChAd3M72 + M72/AS01E	Recombinant live viral vectored	Phase 1	Gong, W., et al. [32]

**Table 3:** Current vaccines in preclinical studies.

Vaccine	Vaccine Type	Description	Author
*M. tuberculosis* H37Rv ΔleuD ΔpanCD strain	Live	Strain used as a TB infection model in *G. mellonella*	Asai, M., et al. [60]
H56:CAF01	Subunit/Adjuvanted	Vaccination strategy of priming and mucosal pull immunization and analysis with uptake of H56/CAF01	Thakur, A., et al. [46]
H64:CAF01	Subunit/Adjuvanted	Protein fusion vaccine	Stockdale, L. & Fletcher, H. [13]
CysVac2/AdVax	Subunit/Adjuvanted	CysVac2 contains Ag85B and CysD; promotes generation of CD4^+^ T cells	Counoupas, C., et al. [61]
BCG-PSN	Subunit/Adjuvanted	BCG polysaccharide and nucleic acid injection	Yan, Q., et al. [62]
LT70	Subunit/Adjuvanted	Induced humoral and cell mediated immunity and higher protective efficacy than BCG	Liu, X., et al. [63]
rBCG:LTAK63	Recombinant	Subunit vaccine with LTAK63 adjuvant; lower levels of LTAK63 created stronger immune response	Carvalho Dos Santos, C., et al. [64]
rBCG(Δ)ais1/zmpl	Recombinant	Recombinant BCG	Yadav, J., et al. [9]
BCG-ZMPI	Recombinant	Recombinant live mycobacterial, lacking *zmp1* gene	Stockdale, L. & Fletcher, H. [13]
FnBPA+ (pValac:ESAT-6)	Recombinant	BCG booster vaccine	Pereira, V.B., et al. [65]
*M. smegmatis* expressing Ag85B	Recombinant	Recombinant bacterium study to evaluate immunogenicity	Kadir, et al. [66]
ChAdOx/MVA PPE15	Recombinant	Viral recombinant	Stockdale, L. & Fletcher, H. [13]
CMV-6Ag	Recombinant	Contains ESAT-6, Ag85A, Ag85B, Rv3407; Rv1733; Rv2626, Rpf A, Rpf C, rpf D	Stockdale, L. & Fletcher, H. [13]
